# DEB-IBM for predicting climate change and anthropogenic impacts on population dynamics of hairtail *Trichiurus lepturus* in the East China Sea

**DOI:** 10.1093/conphys/coac044

**Published:** 2022-07-11

**Authors:** Tao Yang, Qingpeng Han, Harry Gorfine, Xiujuan Shan, Jeffrey S Ren

**Affiliations:** Yellow Sea Fisheries Research Institute, Chinese Academy of Fishery Sciences, 106 Nanjing Road, Qingdao 266071, People’s Republic of China; Function Laboratory for Marine Fisheries Science and Food Production Processes, Pilot National Laboratory for Marine Science and Technology (Qingdao), 1 Wenhai Road, Aoshanwei, Jimo, Qingdao 266200, People’s Republic of China; Yellow Sea Fisheries Research Institute, Chinese Academy of Fishery Sciences, 106 Nanjing Road, Qingdao 266071, People’s Republic of China; School of Biosciences, The University of Melbourne, Parkville 3010, Australia; Yellow Sea Fisheries Research Institute, Chinese Academy of Fishery Sciences, 106 Nanjing Road, Qingdao 266071, People’s Republic of China; Function Laboratory for Marine Fisheries Science and Food Production Processes, Pilot National Laboratory for Marine Science and Technology (Qingdao), 1 Wenhai Road, Aoshanwei, Jimo, Qingdao 266200, People’s Republic of China; Function Laboratory for Marine Fisheries Science and Food Production Processes, Pilot National Laboratory for Marine Science and Technology (Qingdao), 1 Wenhai Road, Aoshanwei, Jimo, Qingdao 266200, People’s Republic of China; National Institute of Water and Atmospheric Research, 10 Kyle Street, P.O. Box 8602, Christchurch 8440, New Zealand

**Keywords:** population model, human pressure, fisheries, environment, DEB model

## Abstract

The hairtail *Trichiurus lepturus* supports the largest fisheries in the East China Sea. The stock has fluctuated in the past few decades and this variation has been attributed to human pressures and climate change. To investigate energetics of individuals and population dynamics of the species in responses to environmental variations and fishing efforts, we have developed a DEB-IBM by coupling a dynamic energy budget (DEB) model to an individual-based model (IBM). The parameter estimation of DEB model shows an acceptable goodness of fit. The DEB-IBM was validated with histological data for a period of 38 years. High fishing pressure was largely responsible for the dramatic decline of the stock in middle 1980s. The stock recovered from early 1990s, which coincided with introduction of fishing moratorium on spawning stocks in inshore waters and substantial decrease of fishing efforts from large fisheries companies. In addition, the population average age showed a trend of slight decrease. The model successfully reproduced these observations of interannual variations in the population dynamics. The model was then implemented to simulate the effect of climate change on the population performance under greenhouse gas emission scenarios projected for 2100. It was also used to explore population responses to changing fishing mortalities. These scenario simulations have shown that the population biomass under SSP1-1.9, SSP2-4.5 and SSP5-8.5 would decline by 7.5%, 16.6% and 30.1%, respectively, in 2100. The model predicts that increasing fishing mortality by 10% will cause 5.3% decline of the population biomass, whereas decrease of fishing mortality by 10% will result in 6.8% increase of the biomass. The development of the DEB-IBM provides a predictive tool to inform management decisions for sustainable exploitation of the hairtail stock in the East China Sea.

## Teaser

DEB-IBM model was developed for population dynamics of Trichiurus lepturus. The model is validated with individual growth and population data. It predicts the reduced population biomass under climate change GHG scenarios. The model simulates fishing mortalities on magnitude change of population biomass.

## Introduction

Fisheries stocks are in decline, and ocean ecosystems are under stress in many parts of the world due to overfishing and destructive anthropogenic forces. Not least of these is climate change, which can lead to large fluctuations in the abundance of fish populations and is contributing to declines in biomass of an increasing number of fish stocks (e.g. Chen *et al.*, 2004; Brander, 2010; Wang *et al.*, 2011). Concurrent pressures of overfishing and climate change have caused considerable decreases in the quality as well as quantity of catches in some important fisheries regions including the East China Sea (Mi, 1997; Chen *et al.*, 2004; Liu *et al.*, 2004; Wang *et al.*, 2011).

The hairtail *Trichiurus lepturus* is widely distributed throughout tropical and temperate waters around the world including northwest Pacific, Indian and Atlantic Oceans (Froese and Pauly, 1996; Sun *et al.*, 2020). It is the most abundant and important demersal fish species in China where it is distributed in the Bohai, Yellow, East China and South China Seas (Du *et al.*, 2020; Kwok and Ni, 2000). There are two major stocks of the hairtail: one in the Bohai and Yellow Seas (called the Yellow Sea stock) and the other in the East China Sea. Some small local stocks are found in the South China Sea and the coastal waters of southern Fujian and Guangdong Provinces (Chen, 1999). China lands 70–80% of the global hairtail catch (Ling *et al.*, 2005; Xu and Chen, 2015), in which the East China Sea stock makes up of 70% of the total catch. Being a demersal species, it is mainly caught by bottom trawling*.* In the 1970s the hairtail stock in the East China Sea was fully exploited with an average annual catch of 0.4 million tonnes, but subsequently dropped to around 0.3 million tonnes by the end of the 1980s. This decline was mainly associated with increasing fishing pressure on spawning stock. Since the introduction of a regulation to protect spawning stock in inshore waters in 1989 (Xu *et al.*, 1994), the stock has recovered considerably. In late 1990s the catch increased to nearly 1 million tonnes (Wang *et al.*, 2011; Xu and Chen, 2015). Although the biomass has fluctuated, total landings have remained at a high level (Wang *et al.*, 2011; Xu and Chen, 2015). Despite its relatively high biomass, population structure and biology have changed. The main changes include a lower population mean age, earlier maturity and a prolonged spawning season (Lin *et al.*, 2006; Ji *et al.*, 2019). These changes in population and biological parameters resulted from the combined effects of heavy exploitation and climate change (Mi, 1997; Wang *et al.*, 2011; Sun *et al.*, 2020). Alteration of population biological processes of the ubiquitous hairtails will inevitably affect the food webs and ecosystems in which they play important roles. Accommodating these factors when assessing the future sustainability of hairtail stocks, requires predictive tools that integrate the energetics of individuals with the performance of their population in responses to climate change and fishing pressure.

Individual-based models (IBMs) have been increasingly used in ecology and anthropology (Wood *et al.*, 2015). An IBM is based on the explicit representation of individuals as unique entities (or actors) that differ from each other and change over their life cycle (DeAngelis and Grimm, 2014). IBMs reflect their broad use by biologists, but these types of models were based on more empirical backgrounds. It is difficult to generalize the outputs from one species to another because IBMs are usually species-specific and differ widely in structure (Grimm *et al.*, 1999). With development of dynamic energy budget (DEB) theory, incorporation of DEB with IBM allows the advantages of using IBMs for generality because DEB theory is a well-tested physiological principle to represent individuals throughout their life cycle. As such, there is an increasing interest in integrating DEB theory with the stochasticity of an IBM to study effects at a population level (e.g. Martin *et al.*, 2012; Goedegebuure *et al.*, 2018). In recent years, DEB-IBMs have been increasingly used to simulate population dynamics of various species (e.g. Beaudouin *et al.*, 2015; Goedegebuure *et al.*, 2018; Bueno-Pardo *et al.*, 2019; Arnould-Pétré *et al.*, 2021).

Ecosystem-based management of fisheries has become one of the important paradigms for contemporary management of fish stocks, requiring an understanding of the population dynamics and associated status of exploited species in the context of environmental variability and human impacts (Ren *et al.*, 2020a). DEB-IBM emerges from the set of DEB parameters of a species and their interaction with environmental variables, which can be used to explore properties of both individual life history traits and population dynamics in response to environmental variability and anthropogenic activities (Grimm *et al.*, 1999). Therefore, a DEB-IBM should provide an efficacious tool for informing decisions about how to manage the exploitation of fish stocks to achieve ecologically sustainable outcomes. Many studies have reported on the hairtail, mostly focusing on age and growth (e.g. Chen, 1999; Zhou *et al.*, 2002; Sun *et al.*, 2020), reproduction (e.g. Li, 1983; Zhang *et al.*, 1983; Kwok and Ni, 1999), population structure (e.g. Wu, 1985; Zhou *et al.*, 2002; Du *et al.*, 2020; Shi *et al.*, 2020) and fisheries (e.g. Xu, 1988; Mi, 1997; Xu *et al.*, 2011). However, development of population dynamic models has not been previously attempted for this species. A DEB-IBM should provide a powerful tool to investigate the impact of climate change and human pressures on fish populations that support viable fisheries.

The purpose of this study was to develop a DEB-IBM model of the hairtail to investigate its energetics of individuals and population dynamics in response to fishing pressures and climate change. Several steps were taken to achieve this goal. First, a DEB model was parameterized with biological data of the East China Sea stock. Second, an IBM was developed and integrated with the DEB model. Third, the model was validated with individual and population data. Finally, the model was implemented for scenario simulations to prospectively investigate the potential effects of climate change and fishing pressure on population dynamics. We anticipate that this modelling study will help inform management decisions about sustainable exploitation of fish stocks in the East China Sea.

## Materials and methods

### DEB model

**Figure 1:**
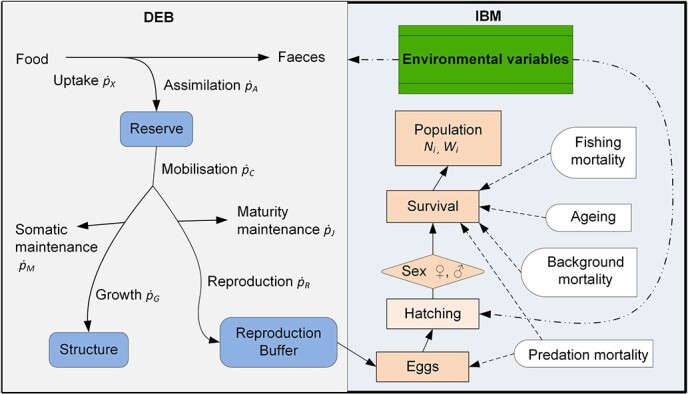
Schematic diagram showing the integration of the individual (DEB) and population (IBM) modules. Individuals undergo development through the DEB model and reproduce. The individuals form the population and undergo population-specific processes (environments and mortalities). Arrows with solid lines donate energy flow. Arrows with dashed lines donate influencing.

A DEB model with metabolic acceleration is used to describe energetics of the hairtail. The energy flow of the model is shown in Fig. 1. The assimilated energy from environment is stored as an energy reserve, which is used for maintenance, structural growth, development and reproduction. The structure of the model is not described here because it is available in Kooijman (2010) and Marques *et al.* (2018). Instead, a brief description of the model is given in Appendix A. We mainly focus on estimation of the model parameters. The parameters of the model were estimated following the AmP procedure (Marques *et al.*, 2018). Parameterization requires zero-variate and univariate data, which were sourced from the literature and listed in Table 1. The former data are a set of single valued trait observations, while the latter data are dependent variables.

**Table 1:** Comparison of observed and predicted zero-variate data. References of observed data and relative error (RE) are specified.

Symbol	Unit	Observation	Prediction	Description	RE	Source
*a_b_*	d	3.29	3.48	Age of birth	0.06	Wan and Meng (2003)
*t_p_*	d	183	205	Time since birth at puberty	0.12	Luo *et al.* (1983)
*a_m_*	d	5475	4933	Life span	0.10	Fishbase
*L_b_*	cm	0.5	0.26	Length at birth	0.48	Chai *et al.* (2015)
*L_p_*	cm	12	16.5	Length at puberty	0.38	Luo *et al.* (1983)
*L_i_*	cm	89.6	68.7	Ultimate length	0.23	Shi *et al.* (2020)
*W_b_*	g	4.25 × 10^−4^	2.8710^−4^	Dry weight at birth	0.32	Luo *et al.* (1983)
*W_p_*	g	80	73.4	Dry weight at puberty	0.08	Luo *et al.* (1983)
*W_i_*	g	5000	5267	Ultimate dry weight	0.05	Fishbase
*GSI*	-	0.04	0.05	Gonado-somatic index	0.18	Kwok and Ni (1999)
*R_i_*	#/d	1192	1323	Maximum reprod rate	0.11	Li (1983), Du *et al.* (1983)
Uni-variate	growth data		d vs cm	Time vs length	0.04	Wu *et al.* (2006); Hong (1980)
Uni-variate	growth data		d vs g	Time vs length	0.07	Wu *et al.* (2006); Hong (1980)
Uni-variate	fecundity		g vs #	Weight vs eggs	0.23	Li (1983), Xu *et al.* (2003)
Uni-variate	Length vs weight		cm vs g	Length vs weight	0.05	Wu *et al.* (2006)

With initial parameter values, the Nelder–Mead method was applied to minimize the loss function (multiplicative symmetric bounded):

(1) \begin{align*} F={\sum}_{i=1}^n{\sum}_{j=1}^{n_i}{w}_{ij}\frac{{\left({d}_{ij}-{p}_{ij}\right)}^2}{{d_i}^2+{p_i}^2}, \end{align*}

where *j* represents the data points in set *n_i_*, *w_ij_* is the weight of dataset, *d_i_* is the mean of the data and *p_i_* is the mean of the prediction. The mean relative error (MRE) and the symmetric mean squared error (SMSE) were computed as goodness-of-fit criteria.

### Population model

The DEB model was coupled to an IBM to form DEB-IBM to simulate an individual as an entity of the IBM (Fig. 1). The development of individuals along their entire life is described by the DEB model. The population is comprised of individuals that are subject to mortality including fishing mortality, density-dependent processes and other environment-dependent mortality. For the population module, individuals are sub-divided into four sub-stages: eggs-larvae, juveniles, females and males. Eggs and larvae have similar attributes and processes and were grouped into one stage. Eggs-larvae change into juveniles at the start of feeding and become females or males at sexual maturity. The number of surviving eggs is a state variable for the eggs-larvae stage. All individuals are described by three state variables: age, generation and survival rate. For juvenile and adult stages, there are additional state variables: length, body weight and sex (female and male). Reproduction is determined by storage of reproductive energy, temperature and spawning period. Individuals born on the same day were assumed to share identical life histories and parameters. All individuals during each spawning period were classified as the same cohort. The population structure in the model is an emergent feature determined by the breeding success and survival of individuals. These dynamics remained stable over time with juveniles being the greatest proportion of the population.

The embryo relies on stored energy supplies (Kooijman, 2010). The age in degree days (*DD*) is an appropriate means for predicting development phases of fish (Neuheimer and Taggart, 2007). The hatching success of eggs depends on age at birth in degree days:

(2) \begin{equation*} {H}_s= \begin{cases}{R}_h:\kern0.5em \mathrm{if}\ DD(t)>=D{D}_{\mathrm{min}}\\ {}0:\mathrm{otherwise}\end{cases}, \end{equation*}

where *R_h_* is the hatching success probability and *DD_min_* is the minimum age in degree days for hatching. The degree day *DD(t)* is calculated as the integral over time (*t*) of temperature above a threshold value:

(3) \begin{equation*} DD(t)=\sum \limits_{i={t}_0}^t\left({T}_i-{T}_{\mathrm{min}}\right)\cdot \varDelta t, \end{equation*}

where *T_i_* is the water temperature, *T_min_* is a lower physiological limit below which hatching does not occur, *∆t* is time from egg to starting of feeding and *t_0_* is the starting time for the development phase. There could be time lag between spawning and starting development of eggs, which can be very short in natural environment. For simplicity, we set *t_0_* = 0.

Five sources of mortality were considered within the DEB-IBM framework. These mortalities are dependent on the fish density, age and environment. Fishing mortality is treated as an external source of mortality. Natural mortality includes four sources: environment-related background mortality (*Z_B_*), density-dependent mortality (*Z_D_*), egg predation mortality (*Z_egg_*) and ageing (*Z_age_*).

Background mortality was related to environmental variables (Wang *et al.*, 2011). Regression analysis between hairtail catches, biomass and environmental variables have shown that the biomass strongly correlated with several environmental variables including rainfall, wind speed and water temperature (Wang *et al.*, 2011). Similarly, recruitment of the hairtail was significantly correlated with river flow and temperature (Liu *et al.*, 2004). Both river runoff and rainfall in this area can affect survival of the hairtail (Chen and Zhu, 1984). Anthropogenic activities may indirectly affect the population of this species. It has been reported that a considerable amount of nutrients was released into the Yangtze River from croplands due to excessive anthropogenic addition of N (Wang, 2006; Zhang *et al.*, 2021). The Yangtze River estuary was identified as the important feeding ground for many fish species including the hairtail (Deng and Zhao, 1991; Chen *et al.*, 1999). Nutrient inputs can cause eutrophication, but may benefit the growth of phytoplankton and zooplankton. River runoff does not only bring inorganic nutrients, but also transports particulate organic matter to the nursery ground of the hairtail (Yang *et al.*, 2015; Kuang *et al.*, 2017). These plankton and organic particles are the main food sources of juvenile hairtails (Chen *et al.*, 1999). In analyses of environmental and mortality data (Xu *et al.*, 2003; Xu *et al.*, 2011; Wang *et al.*, 2011), it has been found that the natural mortality is inversely correlated with rainfall and Yangtze River flow indices, as

(4) \begin{equation*} {Z}_B={M}_{cm}-{M}_{flow}\cdot Flo{w}_{index}-{M}_{rain}\cdot Rai{n}_{index}, \end{equation*}

where *Flow_index_* and *Rain_index_* are Yangtze River flow and rainfall indices, respectively. *Flow_index_* is calculated from annual flow (*flow_i_*) and long-term average annual flow (*flow_mean_*) by $Flo{w}_{index}=( Flo{w}_i- Flo{w}_{mean})/c{f}_{flow}$ with *cf_flow_* as the correction factor (10^12^ m^3^). Similarly, *Rain_index_* is calculated from annual precipitation (*Rain_i_*) and long-term average annual precipitation (*Rain_mean_*) by $Rai{n}_{index}=( Rai{n}_i- Rai{n}_{mean})/c{f}_{rain}$ with *cf_rain_* as the correction factor (1 m).

Cannibalism has been found in many fish species including the hairtail (e.g. Koester and Moellmann, 2000; Beneditto, 2015; Martins *et al.*, 2005; Reuben *et al.*, 1997; Lin *et al.*, 2006). The population density and food resources drive the cannibalism (Lin *et al.*, 2006; Liu *et al.*, 2009). The analysis of stomach contents showed cannibalism as an important feeding behaviour for the adult (Bittar *et al.*, 2012), but the importance of cannibalism showed considerable seasonal variation (Lin *et al.*, 2006). For generalization, we describe density-dependent mortality as a function of fish density, as

(5) \begin{equation*} {Z}_D={M}_d\cdot {B}_{fish}/\left({B}_{fish}+{B}_{fh}\right), \end{equation*}

where *M_d_* is the maximum density-dependent mortality rate, *B_fish_* is fish density and *B_fh_* is half saturation of fish density for density-dependent mortality.

Similarly, studies on diet composition have shown that juvenile hairtail and eggs can contribute up to 50% of stomach content (Lin *et al.*, 2006; Liu *et al.*, 2009). Egg predation mortality (*Z_egg_*) is assumed to depend on density of the fish population and is described as a type-II functional response:

(6) \begin{equation*} {Z}_{egg}={M}_{egg}\cdot {B}_{fish}/\left({B}_{fish}+{B}_{egg h}\right), \end{equation*}

where *M_egg_* is the maximum predation mortality rate, *B_fish_* is fish density and *B_eggh_* is half saturation of fish density for egg mortality.

There is a relationship between energy metabolism and ageing (van Leeuwen *et al.*, 2010). Ageing mortality can be specifically described according to DEB theory (Kooijman, 2010). This mortality generally increases with age and the change in ageing acceleration depends on the Gompertz stress coefficient, Weibull ageing acceleration and body size. However, preliminary simulations have shown that the contribution of ageing mortality to total mortality is considerably small and hence has little effect on population. This is due to low average age of hairtail population. Following Beaudouin *et al.* (2015), we describe ageing mortality (*Z_age_*) to be proportional to age (Age):

(7) \begin{equation*} {Z}_{age}={e}_{age}\cdot {\left( Age- Ag{e}_{\mathrm{min}}\right)}_{+}, \end{equation*}

where *e_age_* is the effect of age on mortality and *Age_min_* is the age threshold of mortality due to ageing.

Fishing mortality (*Z_F_*) is introduced as an external parameter and is estimated from catch and stock abundance data (Mi, 1997; Xu *et al.*, 2003, 2011; Wang and Xu, 2009; Zhang and Chen, 2015; Du *et al.*, 2020). All mortalities are therefore considered as

(8) \begin{equation*} {N}_{t+\varDelta t}={N}_t\cdot {e}^{-\left({\mathrm{Z}}_{\mathrm{F}}+{\mathrm{Z}}_B+{\mathrm{Z}}_D+{Z}_{age}\right)\cdot \varDelta t}, \end{equation*}

where *N_t_* and *N_t + ∆_* are fish abundance at time *t* and *t* + *∆t*, respectively.

### Model setup

**Figure 2:**
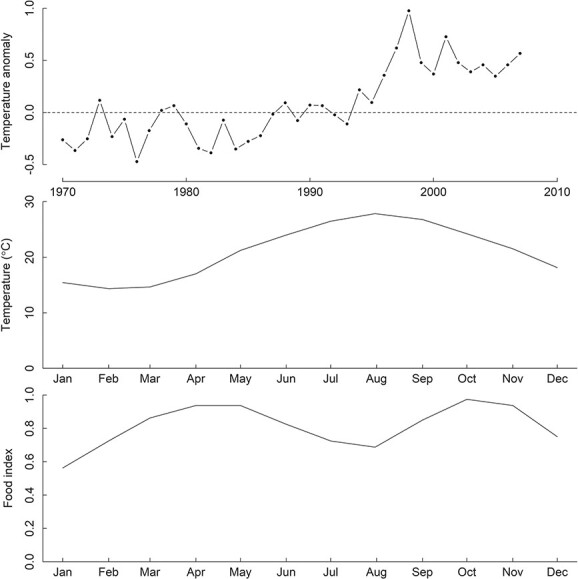
Time-series of environmental variables for model inputs. Sources of data are listed in the text.

The IBM was built with the software Netlogo using the DEB-IBM model developed by Martin *et al.* (2010). The model simulations require large computational resources and time. Because we are constrained by computational resources, population structure and density are not simulated for the whole distribution area. The model was scaled on a 1000-km^2^ area and run with a time step of 1 hour. The East China Sea stock of the hairtail is distributed over a large area of ~5.7 × 10^5^ km^2^ (Cheng *et al.*, 2006; Tang, 2012). The distribution area and depth showed variation with time of the year (Xu and Chen, 2015; Sun *et al.*, 2020), but the majority were distributed within a narrow depth of ~10 m (Zhao, 2005). The abundance data of the adult stock were estimated from fisheries surveys in 1965–2007 (Xu *et al.*, 2003, 2011). For model validation, we converted the total abundance data into density per square kilometre area. For comparison between surveyed and modelled biomass, an average weight of 140 g per individual was used for conversion of numbers of individuals to biomass (Wang and Xu, 2009). At each time step, there are global variables including fishing mortality, spawning period and environmental variables of water temperature, food index and rainfall and river runoff anomaly. The model was set to run for the period of 1965–2007 when environmental and biological data were available. There is some spatial variation of water temperature in the distribution area of the hairtail. The average monthly temperature for the distribution area was used (Fig. 2) (Zhang, 2019). The temperature in the hairtail distribution area has shown inter-annual variation with an increasing trend over the past few decades (Sun *et al.*, 2020) (Fig. 2). A correction to the monthly temperature data was made using anomaly data reported in Sun *et al.* (2020).

The hairtail consumes a wide variety of different organisms. Analysis of stomach contents has shown that the hairtail eats about one hundred prey species belonging to three major groups of pisces, crustacea and cephalopods (Chen and Zhu, 1984; Zhang, 2004; Liu *et al.*, 2009). Overall food availability for the hairtail showed some seasonal variation (Chen and Zhu, 1984; Lin *et al.*, 2006). Food is most abundant in autumn and least abundant in winter months. These studies provided general information for spatial and temporal variation in prey composition, but it is nevertheless not possible to obtain a time-series of food biomass for the hairtail from published data. As an alternative, we compiled biomass information for some of most abundant prey species and constructed a food index based on a weighted sum of the available data (Fig. 2).

Fishing mortality was estimated from total catch for each year and stock abundance at the beginning of the year. We are not able to estimate fishing mortality for each time step of the model. In applying the model, annual fishing mortality was extrapolated within each year.

### Model validation

The DEB model was validated using different datasets from those used for parameter estimation. The growth data were collected from monthly survey data in the East China Sea (Wu, 1985). Length and weight were determined from an examination of otoliths.

The DEB-IBM model validation consisted of a comparison of the model outputs to independent abundance data (Ma, 1989; Xu *et al.*, 2003, 2011; Liu *et al.*, 2004; Wang and Xu, 2009). The population was initialized on 1 January 1965 and the model was run until 2007, with 1965–1969 as a ‘spin-up’ period to stabilize the population. The population variability in the model is generated by the effect of the different environmental variables experienced by the fish born in the spawning period of the year.

### Scenario simulations

The model was used to simulate the impact of climate change and anthropogenic activities on population dynamics of the hairtail. The sea surface temperature is projected to increase in the next century and may rise to 4.5°C by 2100 for the worst greenhouse gas (GHG) emission scenario (SSP5-8.5) (IPCC, 2021). Even under the very low GHG emissions scenario SSP1-1.9, temperatures are assessed to remain elevated by 1.4°C above those of the most recent decade until at least 2100. Fishing pressure is the main contribution to stock size of many fish species including the hairtail (Ma, 1990; Chen, 1999) and anthropogenic activities could be largely responsible for determining the future sustainability of fisheries. To investigate the extent of the effect among different factors, the model is used to explore the effects of climate change and fishing on the hairtail stock. The scenario simulations were based on an assumption that only one targeted variable is changed for each scenario, and the remaining variables are maintained at their present-day condition. The model was run for the same period as the validation for the following five scenarios:

- Sea temperature has risen by 1.4°C of the present-day level in 2100, which reflects the very low GHG emissions scenario (SSP1-1.9).
- Sea temperature has risen by 2.7°C of the present-day level in 2100, which reflects the intermediate GHG emissions scenario (SSP2-4.5).
- Sea temperature has risen by 4.4°C of the present-day level in 2100, which reflects the very high GHG emissions scenario (SSP5-8.5).
- Fishing effort is increased by 10% of the current level,
- Fishing effort is decreased by 10% of the current level.

## Results

### Parameters of DEB model

The parameter estimation shows an acceptable goodness-of-fit, with a low MRE of 0.128 and a low SMSE of 0.143 (Table 2). The ultimate length was underestimated, but the ultimate weight was slightly overestimated. Although there are slight mismatches between data and predictions, the estimation gives good overall predictions for life history and physiological rates.

**Table 2:** DEB and IBM parameter values (parameters in bold symbols are estimated from AmP procedure)

Symbol	Values	Dimension	Definition
DEB parameters			
***z***	5.78	-	Zoom factor relative to reference *Lm* = 1 cm
***ύ***	0.0334	cm d^−1^	Energy conductance
***κ***	0.968	-	Allocation fraction to soma
*κ_R_*	0.95	-	Fraction of reproductive energy fixed in eggs
[$\.{P}$_***M***_***]***	67.46	J cm^−3^d^−1^	Volume-specific maintenance rate
***[E***_***G***_***]***	5240	J cm^−3^	Volume-specific costs for structure
*k̇_J_*	0.002	d^−1^	Maturity maintenance rate coefficient
***δ***_***M***_	0.165	-	Shape coefficient
*T_A_*	8500	K	Arrhenius temperature
*T_H_*	297	K	Higher boundary of the tolerance range
*T_L_*	278	K	Lower boundary of the tolerance range
*T_AH_*	29 000	K	Arrhenius temperature for the rate of decrease at higher boundary
*T_AL_*	11 000	K	Arrhenius temperature for the rate of decrease at lower boundary
***E***^***b***^_***H***_	1.38 × 10^−2^	J	Maturity threshold at birth
***E***^***j***^_***H***_	0.105	J	Maturity at metamorphosis
***E***^***p***^_***H***_	5435	J	Maturity at puberty
$\"{h}$_***a***_	1.12 × 10^−8^	d^−2^	Weibull agieng acceleration
*s_G_*	1.84 × 10^−4^	-	Gompertz stress coefficient
*e_j_*	8500	J g^−1^	Energy content of reserve
*d_V_*	1	g cm^−3^	Specific density of structure
IBM parameters			
*T_min_*	10	°C	Minimum temperature for hatching
*R_h_*	0.90	-	Hatching success probability
*DD_min_*	51.5	°C d	Threshold of degree days for hatching
*M_egg_*	0.083	d^−1^	Maximum daily predation rate of eggs
*B_eggh_*	2.0 × 10^3^	kg km^−2^	Half saturation of fish density for density-dependent mortality
*M_d_*	0.007	d^−1^	Maximum daily predation rate of fish
*B_fh_*	2.2 × 10^3^	kg km^−2^	Half saturation of fish density for density-dependent mortality
*M_cm_*	9.73 × 10^−4^	d^−1^	Coefficient of climate change related mortality
*M_flow_*	4.03 × 10^−4^	d^−1^	Coefficient of Yangtze River flow related mortality
*M_rain_*	6.03 × 10^−5^	d^−1^	Coefficient of rain related mortality
*e_age_*	2.0 × 10^−6^	d^−2^	Effect of age on mortality
*Age_min_*	730	d	Age threshold of mortality due to ageing

### Validation of DEB model

**Figure 3:**
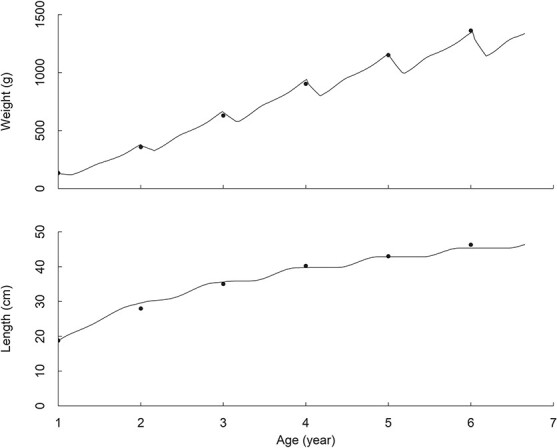
Comparison between observed and modelled growth trajectories of weight and preanal length of the hairtail. Dots are observations and lines are simulations.

The model successfully reproduced the growth of the hairtail (Fig. 3). The simulated length- and weight-at-age of the individual were consistent with observations. The weight showed seasonal variation that reflects variation in temperature, food availability and spawning. Growth in length showed fast increases from spring to autumn, but little growth in winter months. Noticeably, the model predicted that there was hardly any growth in length in some of winter months. This pattern reflects low food variability and temperature.

### Validation of population model

**Figure 4:**
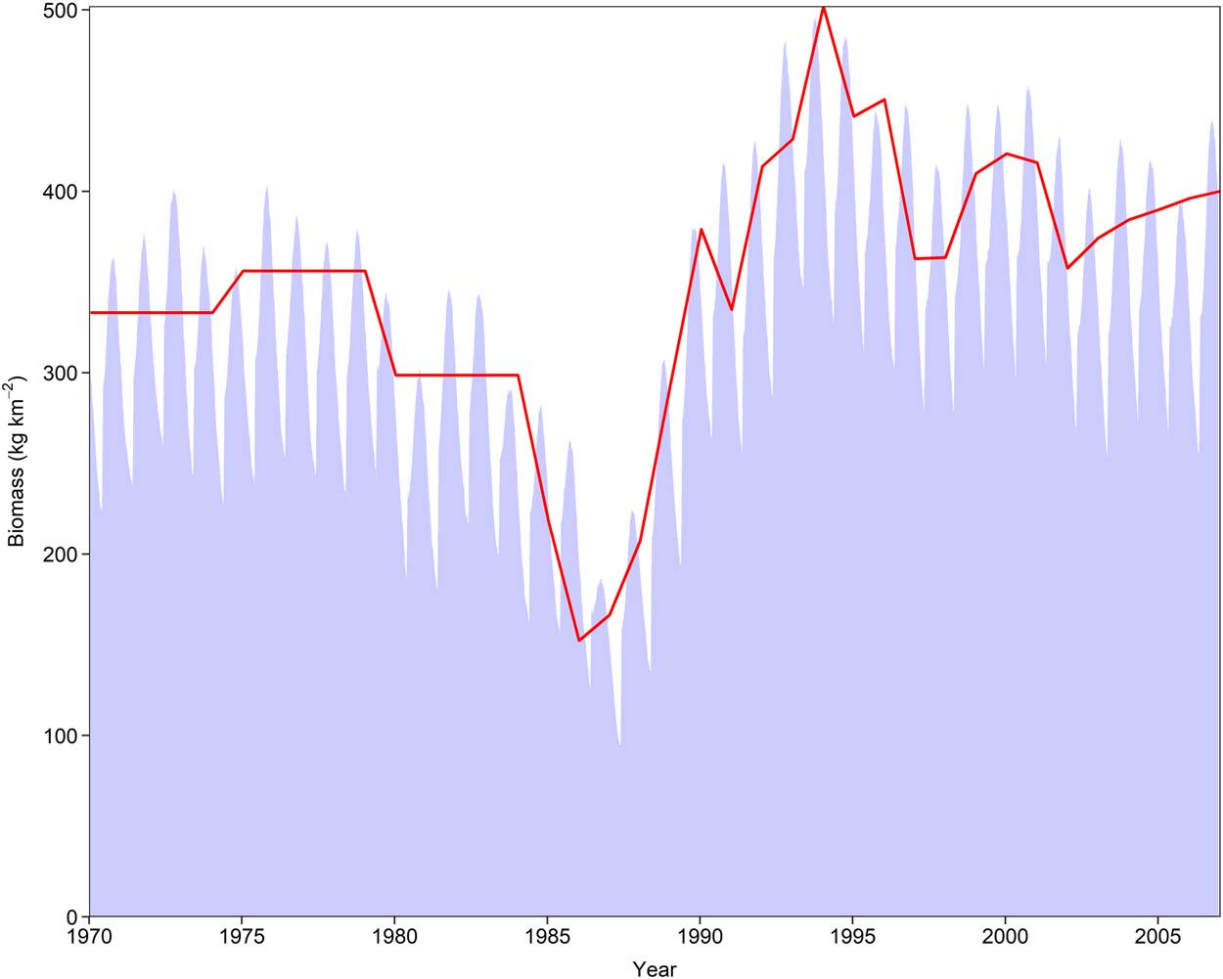
Validation of DEB-IBM simulations for the population biomass of the hairtail in the East China Sea in 1970–2007. The shaded area is the model output, and the line is the observation.

The model can reasonably simulate the inter-annual variation of the population size (Fig. 4). The stock had been in relatively high abundance from the early 1970s to early 1980s. The population then declined considerably from 1984 to its lowest level in 1986. Thereafter, it gradually recovered to another high level in the mid-1990s and remained at this level during the rest of the simulation period. In addition, the seasonal variation reflects both fishing and natural mortalities, with the former contributing the most to both intra- and inter-annual variation.

**Figure 5:**
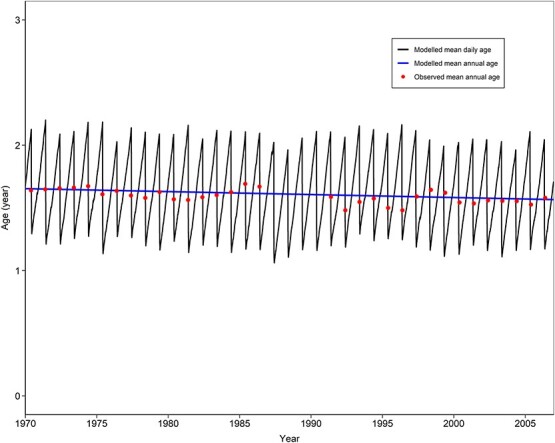
Validation of DEB-IBM simulations for the population mean age of the hairtail in the East China Sea in 1970–2007.

The population mean age is also reproduced (Fig. 5). The modelled average age in 1970–2007 was 1.62 years, which is close to the observation of 1.59 years. The population was dominated by 1-year-old fish. Noticeably, the population age has shown a trend of slight decrease from 1.65 years in 1970 to 1.56 years in 2007 (the blue line in Fig. 5).

### Model predictions for scenarios

**Figure 6:**
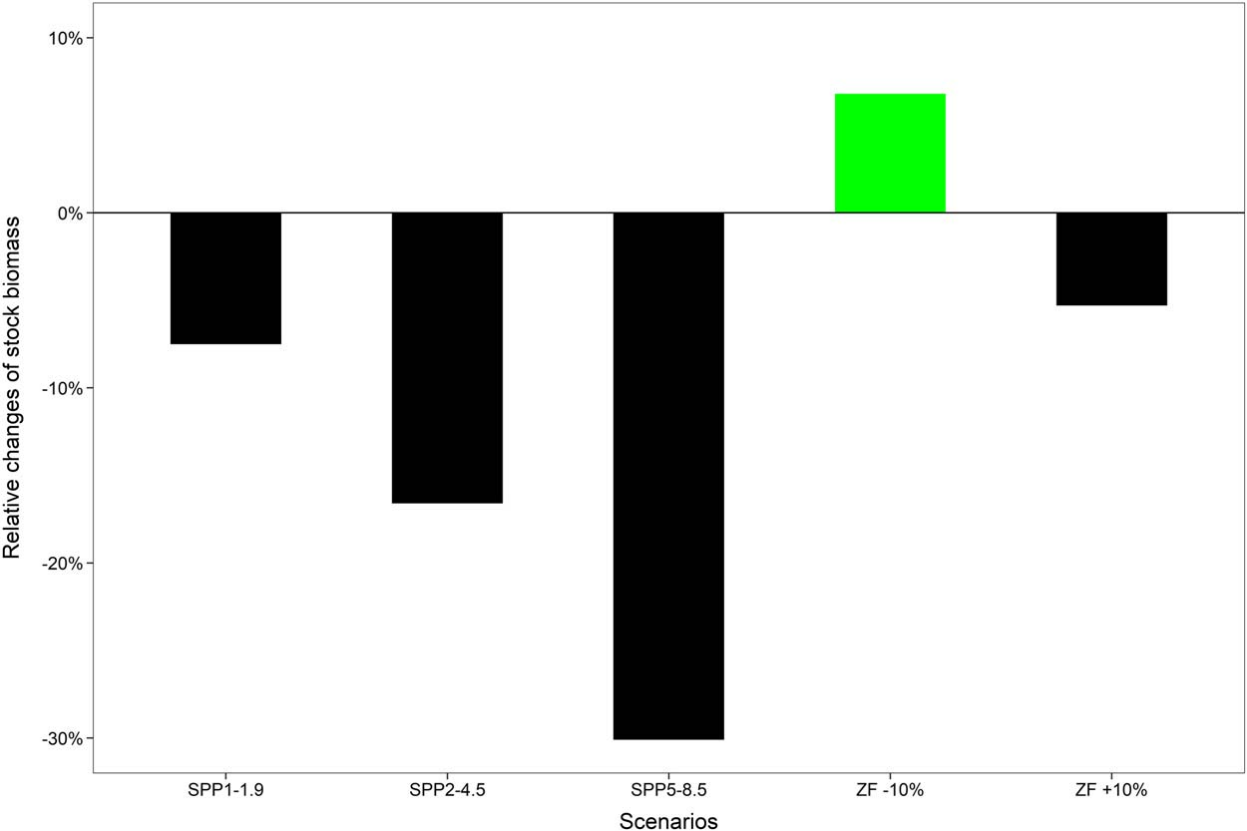
Simulations for percentage changes of population biomass under GHG emission scenarios (SPP1-1.9, SPP2-4.5 and SPP5-8.5) in 2100 and different fishing mortality relative to present-day status. These were based on average values of 38-year simulation results. ZF +10% = fishing mortality increased by 10%. ZF –10% = fishing mortality decreased by 10%.

Scenario simulations have shown that change in temperature will have considerable impact on the population. The model predicts that an increase in temperature would cause significant declines in hairtail population abundance. On average, the biomass under GHG emission scenarios of SSP1-1.9, SSP2-4.5 and SSP5-8.5 in 2100 would be 7.5%, 16.6% and 30.1%, respectively, less than in present-day level (Fig. 6).

Change in fishing mortality will also considerably affect population abundance. The model predicts that increasing fishing mortality by 10% would cause a 5.3% decline in the stock biomass, but a 10% decrease of fishing mortality would enhance the fish stock by 6.8% (Fig. 6).

## Discussion

We have developed a DEB-IBM model that integrates the main ecological factors (e.g. temperature, food dynamics and natural mortality) with human impact. The model is aimed at stimulating the effects of climate change and fishing pressure on the population dynamics of the hairtail. The application of the model has shown that it can reasonably predict the performance of both individuals and population in response to changes in environmental variables and fishing effort. The model provides a predictive tool that will help inform management decisions for sustainable exploitation of the hairtail stock in the East China Sea.

### The model

There is large variation in biological parameters of the hairtail among stocks. This variation has led to considerable differences in DEB parameters. For example, preanal length at age is significantly smaller in the East China Sea than in the coastal waters of India, with a mean length of 18.8 cm for a 1-year-old hairtail in the former and 48.8 cm in the latter (Narasimham, 1976; Hong, 1980; Wu *et al.*, 2006). Similarly, the age-at-first spawning occurs at 1 year in the East China Sea (Ma, 1989; Xu *et al.*, 1997; Sun *et al.*, 2020), whereas it is 2 years for the coastal populations of hairtail in India. Stock-dependent variation in biological variables has been reported in some fish species (e.g. Gatti *et al.*, 2017; Ren *et al.*, 2020a). These differences reflect variation of biological traits of the species among stocks. This variation may be driven by differences in environmental variability between their ecosystems. Evidence showed that fishing pressure and environmental changes have led to evolutionary responses in fish biology for some species (Law, 2000; Heino and Godø, 2002; Ernande *et al.*, 2004). It follows that evolutionary responses may have caused the variation of population characteristics observed among hairtail stocks.

The development of the DEB-IBM model basically follows the concept of DEB-IBM by Martin *et al.* (2010), with some slight modifications, one of which is the exclusion of the maturity variables. We assumed a constant length at birth and puberty. Another difference of our model is the incubation time and maturity variables. Following Beaudouin *et al.* (2015), we have described the incubation time as a function of degree day temperature only. These simplifications do not appreciably compromise the application of the model as previous studies have shown that the uncertainty in model outputs would be insignificant (e.g. Beaudouin *et al.*, 2015). Furthermore, the model is not spatially explicit for the distribution of the population during different life stages. This may have caused some uncertainties in the model predictions. Hairtail migrates for spawning, feeding and wintering at different times of the year (Xu and Chen, 2015), which causes the variability in spatial distribution throughout its life cycle. Incorporation of this DEB-IBM model with a physical–biogeochemical model would potentially improve the predictive capability.

We used food index to represent the seasonal variation in prey biomass. In terms of food supply, there are many studies on prey species of the hairtail (Chen and Zhu, 1984; Zhang, 2004; Liu *et al.*, 2009), but we were unable to construct intra- and inter-annual variation of absolute food density. In addition, seasonal variation in food composition would also be important as prey items vary with life stages (Zhang, 2004). We assumed that the food resource is shared equally among all the individuals, but food resources most likely differ between juveniles and adults. Particulate organic matter and plankton are the main food of juvenile stages. These types of food are abundant in estuaries and coastal areas where the main spawning and juvenile feeding grounds are located (Chen and Zhu, 1984; Sun *et al.*, 2020). We have found that river runoff and rainfall are inversely correlated with natural mortality. Yangtze River is the largest river flowing into the East China Sea. The estuary of Yangtze River is the largest spawning and feeding ground of the hairtail (e.g. Sun *et al.*, 2020). River runoff and rainfall deliver a large amount of particulate organic matter, which is one of the main food sources for the juvenile (Chen *et al.*, 1999). In addition, considerable amount of nutrients flow into the estuary. The nutrients promote phytoplankton growth and hence zooplankton biomass, which provides ideal food for juveniles (Yang *et al.*, 2015; Kuang *et al.*, 2017), whereas fish, crustacea and cephalopods are the main food sources for adults. The inclusion of spatial and temporal variation in prey biomass should be considered for future improvement of the model.

### The model application

The application of the model has shown that it is robust, because the initial number of individuals and the level of the inter-individual variation have relatively little influence on model stability after a 5-year ‘spin-up’ period. The model can reasonably simulate the inter-annual variation of the population in response to environmental variables and fishing mortality during the simulation period of 1970–2007. The characteristics of individual energetics were also reproduced by the model. The scenario simulations have helped assess the potential impact of climate change and fishing pressures on the population. The model simulations showed that rising temperature would cause considerable declines in population abundance. We used the change in temperature as the only variable associated with climate change due to lack of other available data. However, climate change would also cause ocean acidification (OA), which adversely affects physiological activities and survival of larvae/juveniles (Ottersen *et al.*, 2006; Ren *et al.*, 2020b). Previous modelling studies have indicated that OA can reduce growth of shellfish by nearly 30% and reproduction by nearly 20% under climate change scenarios in 2100 (Ren *et al.*, 2020b). Therefore, OA would have further impact on the population. Future experimental studies are required to investigate the effect of OA on physiological responses and survival of the hairtail. Incorporation of this information would help improve the model’s application. In addition, climate change will have indirect effects on marine organisms through food chains, because it will cause a decrease in marine primary production (Frederiksen *et al.*, 2006; Bopp *et al.*, 2013; Kwiatkowski *et al.*, 2017). Climate change may alter the functioning of ecosystems and hence composition of prey species. Climate change directly influences larval survival and recruitment of fishes (Ottersen *et al.*, 2006) and can also affect population dynamics indirectly via ‘top-down’ controls on food web structure. These issues can only be investigated in the ecosystem level. Therefore, further improvement of the model should include other climate change-related variables to predict overall impact on energetics of individuals and the population of the hairtail.

Fishing is largely responsible for declines of many fish stocks (Tang *et al.*, 2016; FAO, 2018), while physical and biological factors also influence population dynamics (Liu *et al.*, 2004; Du *et al.*, 2020). The long-term catch data of the hairtail indicated that the population was largely influenced by both fishing and climate change (Chen *et al.*, 2004; Wang *et al.*, 2011). Our results provide evidence that the inter-annual variability of the hairtail population was mostly driven by fishing and natural mortality. During the simulation period, the hairtail population remained relatively stable from the early 1970s to early 1980s, which indicates that exploitation would have been at an optimum level during this period. However, with increasing fishing effort from the early 1980s onwards, the stock dramatically declined to its lowest level by the late 1980s (Wang *et al.*, 2011). To protect stocks from further decline, a fishing moratorium (i.e. closure of fishing) was implemented during spawning seasons in the late 1980s (Xu *et al.*, 1994; Chen, 1999). Over the same period, fishing effort from large commercial fishing companies has also considerably decreased (Xu *et al.*, 1994; Zhang *et al.*, 2017). Consequently, the stock gradually recovered during the early 1990s and remained at a relatively high level thereafter (Mi, 1997). Interannual variation observed in the hairtail population as described was generally well reproduced by the DEB-IBM model and is consistent with other ecological studies.

For model validation and application, we were unable to simulate the entire population of the hairtail throughout its distributional area due to a lack of computational resources. As an alternative, we ran the model for a more limited area of 1000 km^2^ representative of the population. This simplification may have caused some additional uncertainties in instances where there was large variation in the spatial distribution of the hairtail. Nevertheless, simulating the population at a small scale enabled us to illustrate the potential impact of environmental changes and fishing pressures on population dynamics. The simulation results indicated that the model can generally reproduce population responses to climate change and human effects. This DEB-IBM approach provides a reliable technique that is applicable to other species.

## Conclusion

We have incorporated a DEB model with an IBM to investigate the effects of environmental variability and human pressures on the energetics of individual hairtails and their population dynamics in the East China Sea. Parameterization of the DEB model for the hairtail proved successful as the estimation resulted in low MRE and SMSE. The DEB-IBM validation has shown that the model can reasonably simulate the interannual variation of the hairtail population. The dramatic decline in stock during the late 1980s and subsequent recovery after implementation of management controls were captured by the model. Scenario simulations enabled us to identify the potential trend of the population in response to rising temperature from climate change and changes in fishing effort. However, additional data would help enhance its applicability such as the effect of OA on physiological processes. Information on seasonal and spatial variation in absolute biomass of prey species would also help improve the model predictions. Nonetheless, the present modelling exercise provides a research tool to inform management of the potential magnitude of climate change and human pressures on the fish stocks.

## Funding

This work was supported by National Key R&D Program of China [2018YFD0900906], Taishan Scholars Project of Shandong Province [202103135] and Innovation Team of Fishery Resources and Ecology in the Yellow Sea and Bohai Sea [2021TD01].

## Data availability

Data used in this study were obtained from the literature, which were cited in the text and provided in the reference section.

## A. Appendix A. DEB model

A DEB model (abj-model) with metabolic acceleration is used to describe energetics of the hairtail. An abj-model is applied when acceleration occurs between birth and metamorphosis (Kooijman, 2010; Marques *et al.*, 2018). The isomorphic individual switches to the V1-morphic mode, in which the surface area grows proportionally to structural volume. The metabolic acceleration accommodates change of shape, in which the increase of length is approximately exponential for constant food and temperature. The stage transitions occur when the cumulative investment into maturation (*E_H_*) exceeds certain thresholds: *E_H_^b^* for birth, *E_H_^j^* for metamorphosis and *E_H_^p^* for puberty. At the maturity threshold from birth, an individual reaches its juvenile stage and begins to feed but does not reproduce. Maturity maintenance increases with maturity level until puberty when an individual reaches the adult stage. The energy reserves for reproduction start

**Table A1:** Energy fluxes (processes) and dynamics of the DEB model

Processes	
$c(T)=\exp \Big(\frac{T_A}{T_1}-\frac{T_A}{T}\Big)\cdot {\Big[1+{\mathrm{e}}^{\Big(\frac{T_{AL}}{T}-\frac{T_{AL}}{T_L}\Big)}+{\mathrm{e}}^{\Big(\frac{T_{AH}}{T_H}-\frac{T_{AH}}{T}\Big)}\Big]}^{-1}$	Arrhenius equation
${\dot{p}}_A=\big\{{\dot{p}}_{Am}\big\}\cdot f\cdot {V}^{2/3}\cdot c(T)$	Assimilation
${\dot{p}}_C=E\cdot \frac{\dot{\nu}\cdot \big[{E}_G\big]\cdot {V}^{2/3}+{\dot{p}}_M}{\big[{E}_G\big]+\kappa \cdot E}\cdot c(T)$	Mobilization
${\dot{p}}_M=\big[{\dot{p}}_M\big]\cdot V\cdot c(T)$	Somatic maintenance
${\dot{p}}_J={k}_J\cdot {E}_H\cdot c(T)$	Maturity maintenance
${\dot{p}}_R=\big(1-\kappa \big)\cdot {\dot{p}}_C-{\dot{p}}_J$	Reproduction
${\dot{p}}_G=\kappa \cdot {\dot{p}}_C-{\dot{p}}_M$	Growth
Dynamics	
$\frac{dE}{dt}={\dot{p}}_A-{\dot{p}}_C$	
$\frac{dV}{dt}={\dot{p}}_G/\big[{E}_G\big]$	
$\frac{d{E}_R}{dt}={p}_R\big({E}_H\ge {E}_H^p\big)$	
$\frac{d{E}_H}{dt}={\dot{p}}_H\big({E}_H<{E}_H^p\big)$	
Fish weight	
$W=\frac{E+{k}_R\cdot {E}_R}{e_j}+V\cdot {d}_V$	

to build up in a reproduction buffer being the difference between energy allocation for reproduction and maturity maintenance costs.

The energy uptake from the environment is proportional to food density following a type-II functional response (*f = X/(X + X_k_*), where *X* is food density and *X_k_* is half saturation. The assimilated energy is incorporated into a storage of reserve and subsequently utilized for maintenance, structure growth, development and reproduction. A fixed proportion of the mobilized reserve is allocated to maintenance and growth. The remaining fraction is spent on development in juveniles and reproduction in adults, after subtraction of costs related to maturity maintenance. An individual is described by four state variables which are structure volume (*V*), energy reserve (*E*), reproductive reserve (*E_R_*) and maturity (*E_H_*). The dynamics and intermediate processes are given in Table A1.

## References

[ref80] Arnould-Pétré A A , Guillaumot C, Danis B, Féral J, Saucède T (2021) Individual-based model of population dynamics in a sea urchin of the Kerguelen Plateau (Southern Ocean), *Abatus cordatus*, under changing environmental conditions. Ecol Model 440: 109352. 10.1016/j.ecolmodel.2020.109352.

[ref1] Beaudouin R , Goussen B, Piccini B, Augustine S, Devillers J, Brion F, Péry AR (2015) An individual-based model of zebrafish population dynamics accounting for energy dynamics. PLoS One 10: 1–21. 10.1371/journal.pone.0125841 PMC4418570 25938409

[ref2] Beneditto APMD (2015) What drives the cannibalism of *Trichiurus lepturus* (Linnaeus, 1758) in the coastal area of southeastern Brazil (21-22S)? Int J F Aquat Stud 2: 363–365.

[ref3] Bittar VT , Awabdi DR, Tonini WCT, Vidal MV, Beneditto APMD (2012) Feeding preference of adult females of ribbonfish *Trichiurus lepturus* through prey proximate-composition and caloric values. Neotrop Ichthyol 10: 197–203.

[ref4] Bopp L , Resplandy L, Orr JC, Doney SC, Dunne JP, Gehlen M, Halloran P, Heinze C, Ilyina T, Séférian R et al. (2013) Multiple stressors of ocean ecosystems in the 21st century: projections with CMIP5 models. Biogeosciences 10: 6225–6245.

[ref5] Brander K (2010) Impacts of climate change on fisheries. J Mar Syst 79: 389–402.

[ref6] Bueno-Pardo J , Petitgas P, Kay S, Huret M (2020) Integration of bioenergetics in an individual-based model to hindcast anchovy dynamics in the Bay of Biscay. ICES J Mar Sci 77: 655–667.

[ref7] Chai X , Zhu Y, Wang Y (2015) Study on the embryonic development of *Trichiurus lepturus* in the East China Sea. J Zhejiang Ocean Univ (Nat Sci) 34: 429–432.

[ref8] Chen MH , Wen DJ, Chen CY (1999) Reproduction and estuarine utilization of the grey mullet, *Liza macrolepis* (Smith, 1846), in the Area of Kaohsiung Harbor, Southern Taiwan. Fish Sci 65: 1–10.

[ref9] Chen W (1999) Marine resources, their status of exploitation and management in the People’s Republic of China. FAO Fisheries Circular No. 950, 60 p.

[ref10] Chen Y , Wang F, Bai X, Bai H, Ji F (2004) Relationship between hairtail (*Trichiurus haumela*) catches and marine hydrologic environment in East China Sea. Oceanol Limnol Sin 35: 404–412.

[ref11] Chen Y , Zhu Q (1984) A study on the feeding habit of hairtail fish and the relationship between its food basis and fishing grounds in the Gong Hai (East China Sea). J Fish China 8: 135–145.

[ref12] Cheng JH , Zhang QH, Li SF, Zheng YJ, Li JS (2006) Utilization of Fishery Resources in the Yellow and East China Seas. Shanghai Science and Technology Press, Shanghai, pp. 1–324

[ref13] DeAngelis DL , Grimm V (2014) Individual-based models in ecology after four decades. F1000 Prime Reports 6: 1–6. 24991416 10.12703/P6-39 PMC4047944

[ref14] Deng J , Zhao C (1991) Marine Fishery Biology, Vol. 11. China Agriculture Publishing House, Beijing, pp. 1–163

[ref15] Du J , Chen B, Zhang Q (1983) On the fecundity of hairtail *Trichiurus haumela* (Forskål) in the western Taiwan Strait. Taiwan Strait 2: 122–132.

[ref16] Du P , Chen Q, Li S, Yan Y, Ye W, Yu C (2020) Advances in the *Trichiurus lepturus* changes and habitat driving factors in the East China Sea. J Guangdong Ocean Univ 40: 126–132.

[ref17] Ernande B , Dieckmann U, Heino M (2004) Adaptive changes in harvested populations: plasticity and evolution of age and size at maturation. Proc Biol Sci 271: 415–423. 15101701 10.1098/rspb.2003.2519 PMC1691608

[ref18] FAO (2018) Impacts of climate change on fisheries and aquaculture. FAO Fisheries and Aquaculture Technical Paper 627: 654.

[ref19] Frederiksen M , Edwards M, Richardson AJ, Halliday NC, Wanless S (2006) From plankton to top predators: bottom-up control of a marine food web across four trophic levels. J Anim Ecol 75: 1259–1268. 17032358 10.1111/j.1365-2656.2006.01148.x

[ref20] Froese R , Pauly D (1996) Fish Base-A Biological Database of Fish (Software). ICLARM, Manila.

[ref81] Gatti P , Petitgas P, Huret M (2017) Comparing biological traits of anchovy and sardine in the Bay of Biscay: a modelling approach with the dynamic energy budget. Ecol Model 348: 93–109.

[ref21] Goedegebuure M , Melbourne-Thomas J, Corney SP, McMahon CR, Hindell MA (2018) Modelling southern elephant seals *Mirounga leonina* using an individual-based model coupled with a dynamic energy budget. PLoS One 13: 1–37. 10.1371/journal.pone.0194950 PMC5875804 29596456

[ref22] Grimm V , Wyszomirski T, Aikman D, Uchmanki J (1999) Individual-based modelling and ecological theory: synthesis of a workshop. Ecol Model 115: 275–282.

[ref23] Heino M , Godø OR (2002) Fisheries-induced selection pressures in the context of sustainable fisheries. Bull Mar Sci 70: 639–656.

[ref24] Hong X (1980) A study of the age and growth of the hairtail in the Po Hai and Huang Hai. J Fish China 4: 361–370.

[ref25] IPCC (2021) Summary for Policymakers. Climate Change 2021. In V Masson-Delmotte, P Zhai, A Pirani, SL Connors, C Péan, S Berger, N Caud, Y Chen, L Goldfarb, MI Gomis et al., eds, The Physical Science Basis. Contribution of Working Group I to the Sixth Assessment Report of the Intergovernmental Panel on Climate Change. Cambridge University Press.

[ref26] Ji YP , Liu Q, Liao BC, Zhang QQ, Han YN (2019) Estimating biological reference points for Largehead hairtail (*Trichiurus lepturus*) fishery in the Yellow Sea and Bohai Sea. Acta Oceanol Sin 38: 20–26.

[ref27] Koester FW , Moellmann C (2000) Egg cannibalism in Baltic sprat *Sprattus sprattus*. Mar Ecol Prog Ser 196: 269–277.

[ref28] Kooijman SALM (2010) Dynamic Energy Budget Theory for Metabolic Organisation. Cambridge University Press, Cambridge, p. 490

[ref29] Kuang C , Chen W, Gu J, Su T, Song H, Ma Y, Dong Z (2017) River discharge contribution to sea-level rise in the Yangtze River Estuary, China. Cont Shelf Res 134: 63–75.

[ref30] Kwiatkowski L , Bopp L, Aumont O, Ciais P, Cox PM, Laufkötter C, Li Y, Séférian R (2017) Emergent constraints on projections of declining primary production in the tropical oceans. Nat Clim Chang 7: 355–358.

[ref31] Kwok KY , Ni I-H (1999) Reproduction of cutlassfishes *Trichiurus* spp. from the South China Sea. Mar Ecol Prog Ser 176: 39–47.

[ref32] Kwok KY , Ni I-H (2000) Age and growth of cutlassfishes, *Trichiurus* spp., from the South China Sea. Fish Bull 98: 748–758.

[ref33] Law R (2000) Fishing, selection, and phenotypic evolution. ICES J Mar Sci 57: 659–668.

[ref34] Leeuwen IMM , van Vera J, Wolkenhauer O (2010) Dynamic energy budget approaches for modelling organismal ageing. Philos Trans R Soc B 365: 3443–3454. 10.1098/rstb.2010.0071 PMC2981969 20921044

[ref35] Li C (1983) A study of the individual fecundity and its dynamics of *Trichiurus haumela* (Forskål) of the East China Sea. Oceanol Limnol Sin 14: 220–239.

[ref36] Lin L , Zheng Y, Cheng J, Liu Y, Ling J (2006) A preliminary study on fishery biology of main commercial fishes surveyed from the bottom trawl fisheries in the East China. Mar Sci 30: 21–25.

[ref37] Ling J , Yan L, Lin L, Li J, Cheng J (2005) Reasonable utilisation of hairtail *Trichiurus japonicus* resource in the East China Sea based on its fecundity. J Fish Sci China 12: 726–730.

[ref38] Liu Y , Cheng J, Chen Y (2009) A spatial analysis of trophic composition: a case study of hairtail (*Trichiurus japonicus*) in the East China Sea. Hydrobiologia 632: 79–90.

[ref39] Liu Z , Xu H, Zhou Y (2004) An ameliorative study on the forecast of recruitment stock and catch in winter seasons of hairtail *Trichiurus haumela* in the East China Sea. J Zhejiang Ocean Univ (Nat Sci) 23: 14–18.

[ref40] Luo B , Lu J, Huang S (1983) Maturation of the hairtail *Trichiurus haumela* (Pisces, Trichiuridae). I. The process of maturation and feculiarities of the females. Oceanol Limnol Sin 14: 54–63.

[ref41] Ma Y (1989) A study on the relation between adult stock and recruitment of hairtails (*Trichiurus haumela* Forskål) population in the East China Sea. J Zhejiang Coll Fish 8: 80–85.

[ref42] Ma Y (1990) Fisheries resources and the management of hairtail in the East China Sea. Mar Sci 3: 64–68.

[ref43] Marques GM , Augustine S, Lika K, Pecquerie L, Kooijman SALM (2018) The AmP project: comparing species on the basis of dynamic energy budget parameters. PLoS Comput Biol 14: 1–23. 10.1371/journal.pcbi.1006100 PMC5962104 29742099

[ref44] Martin BT , Zimmer EI, Grimm V, Jager T (2010) DEB-IBM User Manual. Dynamic Energy Budget theory meets individual-based modelling: a generic and accessible implementation. https://www.bio.vu.nl/thb/deb/deblab/debibm/DEB_IBM_manual.pdf, 3, 445, 449, 10.1111/j.2041-210X.2011.00168.x.

[ref45] Martin BT , Zimmer EI, Grimm V, Jager T (2012) Dynamic energy budget theory meets individual-based modelling: a generic and accessible implementation. Methods Ecol Evol 3: 445–449.

[ref46] Martins AS , Haimovici M, Palacios R (2005) Diet and feeding of the cutlassfish *Trichiurus lepturus* in the subtropical convergence ecosystem of southern Brazil. J Mar Biol Assoc U K 85: 1223–1229.

[ref47] Mi C (1997) A study on resources stock structure and variation of reproductive habit of hairtail *Trichiurus haumela* in East China Sea. J Fish Sci China 4: 7–14.

[ref48] Narasimham KA (1976) Age and growth of ribbonfish, *Trichiurus trichiurus* Linnaeus. Indian J Fish 23: 174–182.

[ref82] Neuheimer AB , Taggart CT (2007) The growth degree-day and fish size-at-age: the overlooked metric. Can J Fish Aquat Sci 64: 375–385.

[ref49] Ottersen G , Hjermann DØ, Stenseth NC (2006) Changes in spawning stock structure strengthen the link between climate and recruitment in a heavily fished cod (*Gadus morhua*) stock. Fish Oceanogr 15: 230–243.

[ref50] Ren JS , Jin X, Yang T, Kooijman SALM, Shan X (2020a) A dynamic energy budget model for small yellow croaker *Larimichthys polyactis*: parameterisation and application in its main geographic distribution waters. Ecol Model 427: 109051.

[ref51] Ren JS , Ragg NLC, Cummings VJ, Zhang J (2020b) Ocean acidification and dynamic energy budget models: parameterisation and simulations for the green-lipped mussel. Ecol Model 426: 109069.

[ref84] Reuben S , Vijayakumaran K, Achayya P, Prabhakar RVD (1997) Biology and exploitation of *Trichiurus lepturus* Linnaeus from Visakhapatnam waters. Indian J Fish 44: 101–110.

[ref52] Shi D , Zhang K, Cai Y, Geng P, Xu Y, Sun M, Chen Z (2020) Population structure of *Trichiurus japonicus* in northern South China Sea and parameters of its growth, mortality and maturity. South China Fish Sci 16: 51–59.

[ref53] Sun P , Chen Q, Fu C, Zhu W, Li J, Zhang C, Yu H, Sun R, Xu Y, Tian Y (2020) Daily growth of young-of-the-year largehead hairtail (*Trichiurus japonicus*) in relation to environmental variables in the East China Sea. J Mar Syst 201: 103243.

[ref54] Tang QS (2012) Regional Oceanography of China seas: Fisheries Oceanography. Ocean Press, Beijing, pp. 1–450

[ref55] Tang QS , Ying YP, Wu Q (2016) The biomass yields and management challenges for the Yellow Sea large marine ecosystem. Environ Dev Sustain 1: 175–181.

[ref56] Wan R , Meng Z (2003) The artificial insemination and hatching of *Trichiurus lepturus*. J Fish China 27: 188–192.

[ref57] Wang B (2006) Cultural eutrophication in the Changjiang (Yangtze River) plume: history and perspective. Estuar Coast Shelf Sci 69: 471–477.

[ref58] Wang Y , Jia X, Lin Z, Sun D (2011) Responses of *Trichiurus japonicas* catches to fishing and climate variability in the East China Sea. J Fish China 35: 1881–1889.

[ref59] Wang Y , Xu H (2009) Dynamic analysis on *Trichiurus japonicas* resources in summer closed fishing system in East China Sea. J Zhejiang Ocean Univ (Nat Sci) 28: 384–388.

[ref60] Wood KA , Stillman RA, Goss-Custard JD (2015) Co-creation of individual-based models by practitioners and modellers to inform environmental decision-making. J Appl Ecol 52: 810–815.

[ref61] Wu H , Cheng G, Zhou J, Wang J (2006) Study on the growth of the hairtails *Trichiurus japonicus* in northern China Sea and parameters and its growth, mortality and maturity. South China Fish Sci 16: 51–59.

[ref86] Wu J (1985) Age and growth of *Trichiurus lepturus* on the off-shore fishing ground of Zhejian Province. J Zhejiang Coll Fish 4: 9–23.

[ref62] Xu H , Liu Z, Ding Y, Xu Y (1994) Resource condition and management countermeasure of largehead hairtail in the East China Sea. J Zhejiang Coll Fish 13: 6–11 DOI: CNKI:SUN:REEF.0.1994-01-001.

[ref83] Xu H , Li Z, Zu Y (1997) Comment on hairtail stock dynamic and discussion on management situation. J Zhejiang Coll Fish 16: 219–225.

[ref63] Xu H , Liu Z, Zhou Y (2003) Variation of *Trichiurus haumela* productivity and recruitment in the East China Sea. J Fish China 27: 322–327.

[ref64] Xu H , Liu Z, Zhou Y, Wang Y (2011) The relation between parents and recruitment of hairtail on status of summer closed fishing in East China Sea. Fish Modern 38: 64–69.

[ref65] Xu Y (1988) Characteristics of hairtail fish (*Trichiurus haumela*) population and fishery management in the East China Sea. J Ecol 7: 4–8.

[ref66] Xu Z , Chen J (2015) Migratory routes of *Trichiurus lepturus* in the East China Sea, Yellow Sea and Bohai Sea. J Fish China 39: 824–835.

[ref67] Yang SL , Xu KH, Milliman JD, Yang HF, Wu CS (2015) Decline of Yangtze River water and sediment discharge: impact from natural and anthropogenic changes. Sci Rep 5: 12581. 26206169 10.1038/srep12581 PMC4648474

[ref68] Zhang B (2004) Feeding habits and ontogenetic diet shift of hairtail fish (*Trichiurus lepturus*) in East China Sea and Yellow Sea. Mar Fish Res 25: 6–12.

[ref69] Zhang C (2019) Spatial and temporal variations of sea surface temperature in the East China Sea and west Pacific Ocean in last 140 years. Thesis of Master of Science. Normal University of Shanghai, 69 p.

[ref70] Zhang K , Chen Z (2015) Using Bayesian state-space modelling to assess *Trichiurus japonicus* stock in the East China Sea. J Fish Sci China 22: 1015–1026.

[ref74] Zhang J , Chen B, Zhang Q (1983) On the fecundity of hairtail, *Trichiurus haumela* (Forskål) in the western Taiwan Strait. Taiwan Strait 2: 122–132.

[ref71] Zhang Q , Hong W, Chen S (2017) Stock changes and resource protection of the large yellow croaker (*larimichthys crocea*) and ribbon fish (*Trichiurus hapanicus*) in coastal waters of China. J Appl Oceanol 36: 438–449.

[ref72] Zhang Y , Wu H, Yao M, Zhou J, Wu K, Hu M, Shen H, Chen D (2021) Estimation of nitrogen runoff loss from croplands in the Yangtze River Basin: a meta-analysis. Environ Pollut 272: 116001. 33187836 10.1016/j.envpol.2020.116001

[ref85] Zhao X (2005) In situ target-strength measurement of young hairtail (*Trichiurus haumela*) in the Yellow Sea. ICES J Mar Sci 63: 46–51.

[ref73] Zhou Y , Xu H, Liu Z, Xue L (2002) A study on variation of stock structure of hairtail *Trichiurus haumela* in the East China Sea. J Zhejiang Ocean Univ (Nat Sci) 21: 314–320.

